# JESA in the time of COVID

**DOI:** 10.1007/s40881-021-00113-9

**Published:** 2021-12-14

**Authors:** Maria Bigoni, Dirk Engelmann

**Affiliations:** 1 University of Bologna, Bologna, Italy; 2 Humboldt-Universität zu Berlin, Berlin, Germany

This is not what we had planned as our first editorial. We had envisioned that in the first or second issue after we took over as editors of JESA, we would include an editorial where we would lay out the plans we had to make JESA an even more attractive outlet. Instead, just a few weeks after we started, as many others we were affected by the simultaneous increase of teaching effort due to the switch to the online mode, school closures, and irregular child care. Thus, from early on, we barely managed to keep track with daily business, in times accumulating large backlogs of papers to process and our first editorial appears closer to the end of our term, which is due in 2022, than to its beginning.

December 2021 is a good time to write this Editorial, however. This issue of JESA appears exactly 2 years after the onset of the COVID-19 pandemic, and the world is not out of it yet. Still, we can try to take a step back and look at the consequences that this massive, unpredictable shock had not only on society as a whole, but specifically for our discipline, experimental economics. The report on “Publication trends in experimental economics” by Ernesto Reuben and co-authors, to appear in the next issue of JESA, illustrates how in recent years, top general interest journals devoted progressively less attention to traditional lab experiments with student subjects, as compared to other types of experiments, including field and online experiments. The reasons behind this tendency are not obvious, but we have the impression that there is a demand for a tighter link between the experiment and the “real world”, and for experiments that have more direct policy implications.

We believe that the pandemic has the potential to be a game changer in this context. On the one hand, it generated opportunities to look at old problems—like the sensitivity of preferences and norms to exogenous shocks—from a new perspective, and it also inspired novel research questions with a clear policy relevance, which can be usefully addressed relying on the clean and sound methodology of experimental economics. On the other hand, it raised new methodological challenges which have been promptly addressed with the introduction of several important innovations.

A set of excellent examples of this is assembled in the Special issue “Experiments in the time of COVID-19 – challenges and insights” that comprises most of this issue. Research questions that were inspired by the pandemic are addressed by Abdelaziz Alsharawy and co-authors, Yiting Guo and co-authors, Gyuala Seres and co-authors, as well as by Fatih Sonmez. Abdelaziz Alsharawy and co-authors investigate in a repeated cross-section in MTurk how fear of COVID-19 in the early phase of the pandemic correlates with economic preferences, finding plausible relations to some but not all variables of interest. This article is also of methodological interest, calling for care when running experiments in unusual times such as a pandemic. Yiting Guo and co-authors study in an online experiment how watching videos of leaders calling for action or volunteers early in the pandemic in China affects pro-social and risk-taking behavior. This study nicely complements the one by Abdelaziz Alsharawy and co-authors with behavioral rather than survey data and not fear but inspiration being the determinant. Gyula Seres and co-authors study in a field experiment how wearing face masks before it was mandatory affected the attention to recommended distances in queues outside of shops. In contrast to the risk-compensation hypothesis, wearing a face mask increases the distance that other people keep. This article made use of the novel situation to provide a clean test of risk compensation. Finally, Fatih Sonmez provides a conceptual replication of a previous study that had shown that evoking thoughts about death makes people more patient. In his study, he observes the opposite effect, with participants being primed to think about COVID-related death becoming less patient, possibly because in this situation, thinking about death is not very abstract anymore but rather a concrete thought.

Methodological contributions come from Jiawei Li and co-authors, Buso and co-authors, as well as from Harrison and co-authors. Jiawei Li and co-authors compare both subject demographics and behavior in a laboratory sample and two online samples using a combination of z-Tree unleashed and Zoom, one with webcam off and one with webcam on. Reassuringly for experimenters, the online samples do not deviate much from the laboratory sample, though with the webcam-off sample being more noisy. The main result is confirmed by Buso and co-authors, who illustrate how to conduct an experiment with real-time interaction among participants on oTree, combined with Cisco-WebEx. The article by Harrison and co-authors complements the previous two by illustrating how oTree offers the possibility of running cross-country experiments with hundreds of participants per session, which would have been unimaginable with a standard laboratory approach. All these three contributions are based on experiments involving standard student subjects; the methodological advancements they describe, however, can be adopted also with a more diverse sample of participants. This is even more true now that the virus containment measures introduced around the globe forced most of the working-age populations to get used to online communication and payment platforms. Harrison and co-authors conclude their article saying that “COVID-19 emerged as a mother of invention,” and we agree. The technical innovations that have been introduced during the past 2 years can be usefully combined with field interventions and natural experiments, to adopt a rigorous experimental methodology in the study of questions that before could only be addressed by means of surveys and secondary source data.

With a higher share of methodological contributions and replications than a usual issue along with papers addressing known questions in a novel setting, this special issue nicely illustrates where we would like JESA to go in the future to make it a more valuable outlet for both authors and readers. We have worked on plans for increasing the attractiveness of replications, but implementing them will likely fall on our successors.

Along with the COVID-19 special issue, this issue contains a report prepared by Lionel Page and co-authors on the replication crisis, which among other solutions stresses the value of replications and suggests ways to make them more attractive to conduct. This gives us the opportunity to announce the new data-policy we are phasing in at JESA, consistent with procedures at Experimental Economics. Starting from 2022, authors of papers accepted for publication in JESA will be asked to provide detailed replication files, including the original dataset, the programs used to clean and analyze the data, and any programs used to collect experimental data. The final paper included in this issue by Peter Moffat and Graciela Zevallos-Porles provides a nice methodological contribution on the treatment of corner solutions in dictator games.

While our turnaround statistics have generally been dreadful, we managed to process the papers for the COVID-19 special issue without too much delay and publish them a year after the submission deadline. This was possible due to some of the authors managing to provide a second-round minor revision within a week and in one case a reviewer who provided a careful report on a revision within a day. We are extremely grateful for this help in getting the special issue published this year. Unfortunately, enabling the publication of the special issue in the December issue meant that we let some of the papers jump the queue, which further delayed the process of some regular submissions.

Thus, we have to return to the topic of the beginning of this editorial and apologize to the authors of the regular papers that had to wait even longer than they would have anyway due to our decreased processing speed because we wanted to publish the COVID-19 special issue as early as possible as related research is progressing quickly. It turned out to be an unfortunate choice to pick two editors with relatively heavy teaching loads and relatively young children. ESA was clearly not planning for a pandemic, as (almost) nobody else was. We could see the effect on others as well, as it proved harder than usual to find reviewers and some reviewers and authors took rather long to provide reports and revisions. The main responsibility for the slow process, however, falls on us. We dare to close with one remark on statistics, though. When we took over, we agreed that the statistic we care most about was complaints after decisions. We still stand at zero. As long as we handle papers for JESA, we will do our best to keep it at that.

